# A Case of *NAA10*-related Syndrome With Prolonged QTc Treated With a Subcutaneous Implantable Cardioverter Defibrillator After Ventricular Fibrillation

**DOI:** 10.1016/j.cjcpc.2022.10.001

**Published:** 2022-10-07

**Authors:** Yuta Mizuno, Yasuhiro Ichikawa, Shun Kawai, Takuya Wakamiya, Hiroaki Murakami, Kenji Kurosawa, Hideaki Ueda

**Affiliations:** a Department of Cardiovascular Medicine, Kanagawa Children’s Medical Center, Yokohama, Japan; b Department of Pediatrics, Yokohama City University Hospital, Yokohama, Japan; c Division of Medical Genetics, Kanagawa Children’s Medical Center, Yokohama, Japan

## Abstract

NAA10 is an enzyme involved in the N-terminal acetylation of proteins. *NAA10*-related syndrome is caused by a pathogenic variant of *NAA10* on X chromosome, resulting in several phenotypes, including mental retardation, hypotonia, growth retardation, and various external malformations, with varying degrees of severity. With regard to cardiac diseases, hypertrophic cardiomyopathy is a possible complication. Some mutations are also associated with long QT syndrome. Herein, we describe the case of a 7-year-old boy with a novel *NAA10* mutation who experienced cardiopulmonary arrest possibly due to long QT syndrome and was implanted with a subcutaneous implantable cardioverter defibrillator.

## Résumé

La NAA10 est une enzyme qui intervient dans l’acétylation N-terminale des protéines. Le syndrome lié au gène *NAA10* est causé par un variant pathogène du *NAA10* sur le chromosome X qui entraîne plusieurs phénotypes, comme une déficience intellectuelle, une hypotonie, un retard de croissance ou différentes malformations externes, et ce, à divers degrés de sévérité. En ce qui concerne les maladies cardiaques, une cardiomyopathie hypertrophique est une complication possible. Certaines mutations sont également associées au syndrome du QT long. Nous décrivons ici le cas d’un garçon âgé de sept ans qui présente une nouvelle mutation du gène *NAA10* et qui a fait un arrêt cardiorespiratoire, possiblement en raison d’un syndrome du QT long. L’enfant a reçu un défibrillateur cardiaque implantable sous-cutané.

N-terminal acetylation is one of the most common protein modifications in eukaryotes.^1^ NatA, a major N-terminal acetyltransferase complex, comprises a catalytic subunit encoded by *NAA10* (Xq28) and an auxiliary subunit encoded by *NAA15* (4q31.1).^2^ In 2011, an *NAA10* variant (c.109T>C:p.Ser37Pro) was identified as the cause of an X-linked recessive lethal disorder called Ogden syndrome.^3^ Since then, several novel *NAA10* variants have been described. Currently, a wide variety of phenotypes are associated with *NAA10*-related syndrome, including developmental and intellectual disabilities, cardiac diseases, growth disorders, and external malformations. Cardiac diseases include hypertrophic cardiomyopathy (HCM)^4^ and long QT syndrome (LQTS).^5^ We report the case of a 7-year-old boy with a novel hemizygous *NAA10* mutation and LQTS who suffered from cardiac arrest and underwent subcutaneous implantable cardioverter defibrillator (s-ICD) implantation.

## Case

**Figure 1:**
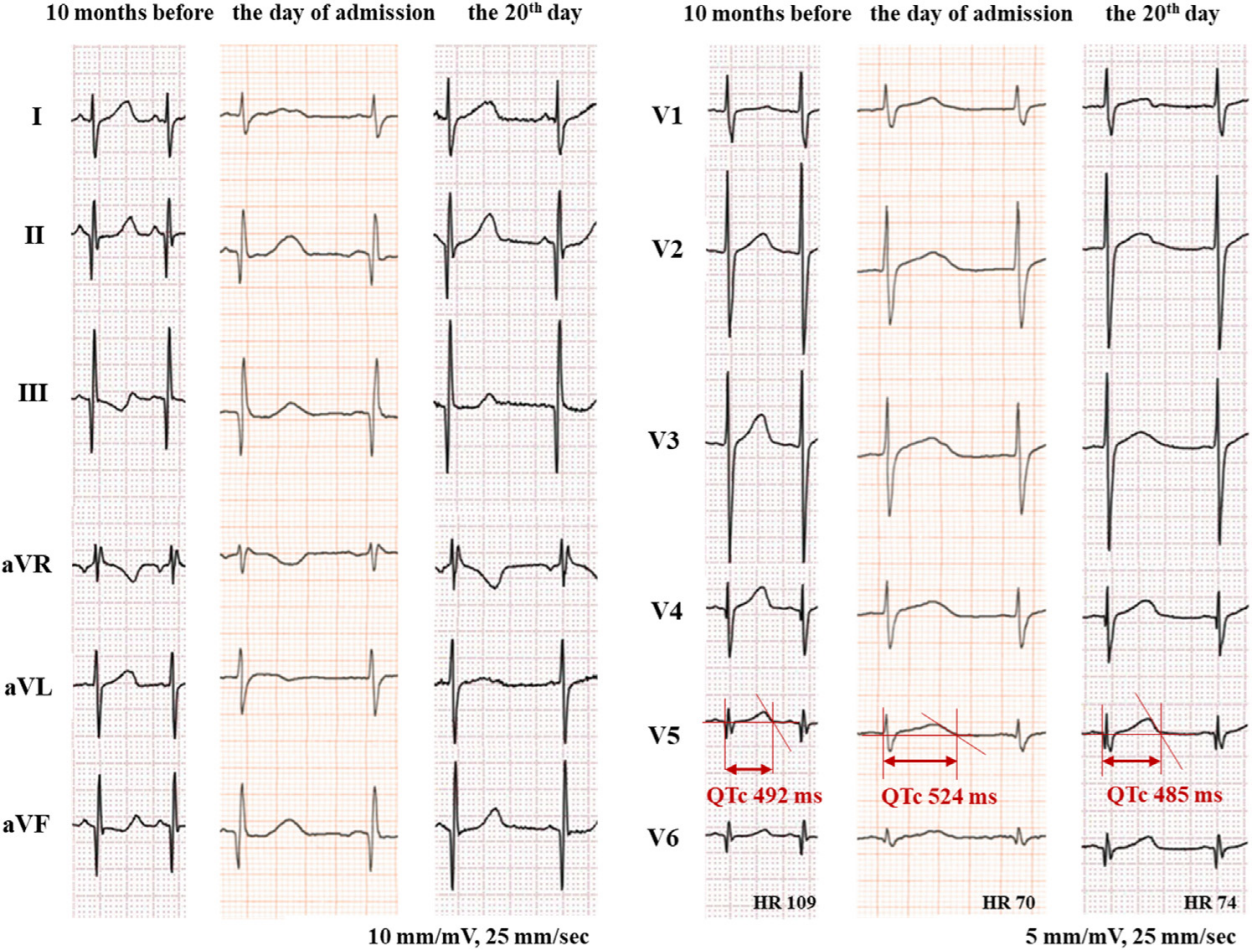
Electrocardiogram performed 10 months before the cardiac arrest, on the day of admission, and on the 20th day of admission. Each electrocardiogram shows a QTc of 492 milliseconds, 524 milliseconds, and 485 milliseconds, respectively (Bazett). HR, heart rate; QTc, corrected QT interval.

The patient was born to a Caucasian father and a Japanese mother, both of whom were healthy, and he was their first child. Mild developmental impairment, autism, and gait abnormality were noted around the age of 4 years. The child also presented with mild HCM at the age of 6 years, which was a year before the cardiac arrest. Concentric left ventricular hypertrophy was noted. The maximum left ventricular (LV) wall thickness in end-diastole was 11.8 mm at the basal anteroseptal segment. The interventricular septum diameter in end-diastole was 9.6 mm (*z* score = 2.0), and the LV posterior wall thickness in end-diastole was 8.3 mm (*z* score = 1.8). There was no evidence of LV outflow tract stenosis (LVOTS) or left atrial dilation. There had been no previous arrhythmia, syncope, or signs of heart failure. The corrected QT interval (QTc) measured during the initial visit was 450 milliseconds (Bazett), but it was abnormal during the follow-up outpatient consultations (QTc ≥ 480 milliseconds) (Fig. 1). Genetic testing was performed for better assessment of HCM. A novel mutation in *NAA10* (NM_003491.3:c.278A>G:p.Gln93Arg) was identified via next-generation sequencing using the TruSight One Sequencing Panel (Illumina, Inc, San Diego, CA), which covers most of the major genes of clinical relevance including LQTS-related genes. The same mutation was identified in his asymptomatic mother, although in the heterozygous state. As for gait abnormality, he had a dragging gait in the left sole with his left knee joint extended. Occasionally, his left hand got stiff while playing with toys. The boy seemed to exhibit symptoms of left-sided Parkinsonism, which could be associated with the *NAA10* mutation.

At the age of 7 years, the patient collapsed on his way back from the bathroom at a parking lot and developed pallor, with his eyes rolled back, and tonic-clonic seizures. The passengers in the car immediately started chest compressions. When the emergency team arrived, approximately 4 minutes after the patient collapsed, he had stopped breathing. Ventricular fibrillation (VF) was detected on an electrocardiogram (ECG) monitor, and defibrillation was performed (Supplemental Fig. S1). Subsequently, the patient showed return of spontaneous circulation approximately 7 minutes after he collapsed. When he was transported to the previous hospital, blood circulation was maintained without the need for ionotropic drugs. Given the prolonged disturbance in consciousness, he was intubated and transported to our hospital.

His body weight was 27 kg (−0.1 standard deviation) and height was 120 cm (1.1 standard deviation). His vital signs were as follows: body temperature, 37.7°C; heart rate, 104/min; respiratory rate, 16/min; blood pressure, 127/79 mm Hg; and saturation, 100% (O_2_, 6 L/min, intubated). On physical examination, Levine II/VI systolic ejection murmur was heard at the upper-middle left sternal border. Blood tests revealed neither acidosis nor cardiac enzyme elevation. Electrolyte levels were normal. ECG performed at the time of admission showed QTc prolongation of 524 milliseconds (Fig. 1).

He was extubated on the fourth day after induced normothermia. Head magnetic resonance imaging revealed no ischemic changes. He recovered without neurological sequelae.

**Figure 2:**
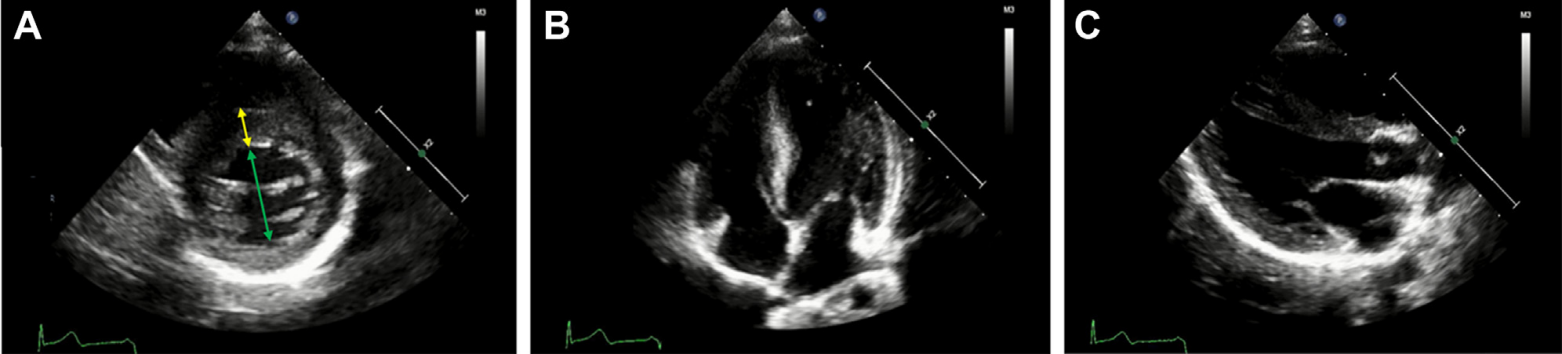
Echocardiography performed on the 14th day of admission, which revealed similar findings to that performed on the day of admission; maximum intraventricular septum, 13 mm (**yellow double-sided arrow**) and left ventricular end-diastolic diameter, 33 mm (**green double-sided arrow**). There was no evidence of left ventricle outflow tract stenosis; the peak velocity was 1.5 m/s. No signs of systolic anterior movements of the anterior mitral leaflet were observed. (**A**) Parasternal short-axis view at end-diastole; (**B**) apical 4-chamber view at end-diastole; (**C**) parasternal long-axis view at end-diastole.

Given that apart from LQTS, HCM is also considered a cause of VF,^4^ we needed to assess the severity of HCM. ECG revealed no signs of left ventricular hypertrophy, including tall R waves, ST-T changes, or inverted T waves in left precordial leads (Fig. 1). The echocardiogram revealed no structural anomaly in addition to concentric HCM. It showed neither LVOTS nor systolic anterior movements in the anterior mitral leaflet (Fig. 2). Delayed contrast-enhanced cardiac magnetic resonance imaging performed on the 13th day revealed no myocardial fibrosis. With regard to LQTS, an ECG performed 10 months before the cardiac arrest revealed a QTc of 492 milliseconds. Because acute events such as the loss of consciousness or seizures can temporarily increase the QT interval, ECGs were repeated. The QTc measured on the 20th day was 485 milliseconds (Fig. 1). The presence of an LQTS risk score of ≥3.5 points without any secondary causes for QTc prolongation (4 points: QTc ≥ 480 milliseconds, syncope without stress) suggested a high probability of LQTS.^6^ Holter ECG on the 11th day showed no arrhythmia. The administration of nadolol (15 mg, once daily) was commenced on the 14th day of admission. Low-dose nadolol (0.5 mg/kg/d) was administered to avoid bradycardia. The patient was transferred on the 22th day for the implantation of an s-ICD; the shock zone (VF zone) and conditional zone (ventricular tachycardia zone) were set at 250/min and 230/min, respectively. The nadolol dose was increased to 30 mg once daily 10 months later, but no bradycardia was observed. Two years have passed since the implantation, and no s-ICD activation has occurred.

## Discussion

We report the case of a 7-year-old boy with a novel *NAA10* missense variant (NM_003491.3:c.278A>G:p.Gln93Arg) who developed cardiac arrest. He exhibited QTc prolongation. He also had mild developmental impairment, autism, gait abnormality, and mild HCM. Patients with *NAA10*-related syndrome exhibit various phenotypes with varying degrees of severity, depending on the mutation. Because *NAA10* is thought to acetylate over 8000 different proteins, each disease mutation may affect the acetylation of the different sets of substrates, resulting in different phenotypic features.^5^ Casey et al.^5^ mentioned that only patients with mutations in exons 2-6 of *NAA10*, where the N-acetyl transferase domain exists, experienced cardiac arrhythmias. Conversely, the mutation in patients without arrhythmia was found to exist outside the N-acetyl transferase domain. This observation suggests a possible interaction between the *NAA10* N-acetyl transferase domain and a known or novel arrhythmia gene. Mutations in this domain may reduce the activity of N-terminal acetyltransferase, thereby affecting the regulation of gene expression related to arrhythmogenesis. The novel mutation in our report (NM_003491.3:c.278A>G:p.Gln93Arg) is located in exon 5. Considering Casey’s report,^5^ our novel mutation may be linked to arrhythmogenesis, which is consistent with the patient’s clinical symptoms. Further studies are warranted to clarify the genotype-phenotype relationship and identify the *NAA10* gene region involved in the arrhythmia, which could aid in the risk stratification of patients with arrhythmia onset, including sudden cardiac death (SCD).

In the present case, the precise cause of VF was unknown. The pre- and postarrest ECG showed a QTc of ≥480 milliseconds with the episode of syncope and arrest, indicating a high probability of LQTS. However, we were unable to fully determine whether the cause of VF was LQTS. Because of the patient’s gait abnormality, an exercise stress test could not be performed. An epinephrine QT stress test was also considered but was not performed owing to safety concerns. We also considered HCM as the cause of VF; however, the HCM was mild and felt to be less likely to cause VF considering the absence of LVOTS or myocardial fibrosis, mild LV wall thickening, and no previous history of syncope. There have been some studies involving paediatric patients with HCM for SCD prediction models, such as “HCM Risk-Kids,” which uses 5 predictor variables (unexplained syncope, maximum LV wall thickness, left atrial diameter, LV outflow tract gradient, and nonsustained ventricular tachycardia).^7^ Based on the data of 10 months before the arrest, this case was categorized as low risk (<4% estimated SCD risk at 5 years). However, because these scores have imperfect sensitivity and specificity for predicting life-threatening events, we cannot rule out the possibility that HCM is the cause of VF. In conclusion, although we speculated that LQTS may have caused VF, the precise cause could not be determined.

Despite the scarcity of data for children, ICD interventions for terminating life-threatening arrhythmias were common in a high-risk paediatric HCM cohort, such as those who experienced resuscitated cardiac arrest or sustained ventricular tachycardia.^8^ In this case, ICD was implanted for the secondary prevention of cardiac arrest. With regard to the types of ICDs, a transvenous ICD is difficult to use for patients weighing only 27 kg, and open thoracic surgery is more invasive. For such young patients who would likely require life-long ICD therapy but not pacing therapy, s-ICD may provide an advantage in avoiding transvenous lead-related risks. Therefore, s-ICD was chosen this time.

**Novel Teaching Points**

• In the treatment of *NAA10*-related syndrome, it is necessary to recognize the possibility of LQTS or HCM development and to pay attention to SCD.
• In recent years, progress has been made in identifying the *NAA10* mutation sites associated with arrhythmogenesis. It is important to accumulate more information and aim for risk stratification of patients with severe arrhythmias.
• The s-ICD should be considered for young patients with a lifetime risk of SCD, including *NAA10*-related syndrome with prolonged QTc.
