# Supplementary figures and images for: A Case of NAA10-related Syndrome With Prolonged QTc Treated With a Subcutaneous Implantable Cardioverter Defibrillator After Ventricular Fibrillation
Source: CJC Pediatr Congenit Heart Dis. 2022 Oct 7;1(6):270–3. doi: 10.1016/j.cjcpc.2022.10.001 (PMC10642139; doi:10.1016/j.cjcpc.2022.10.001)

1 **Supplemental Fig. S1**

2 Ventricular fibrillation detected on the electrocardiogram monitor.

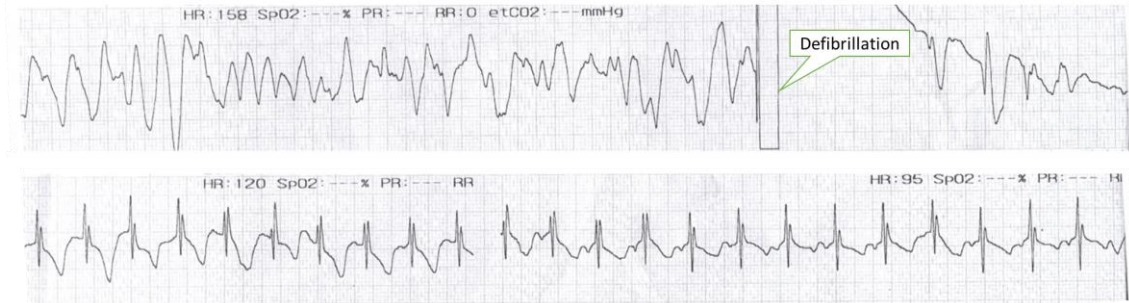

3

4

Supplement: Supplemental Fig. S1 [file mmc1.pdf]
